# Obesity and Outcomes in Adoptive Cellular Therapy in Solid Tumors

**DOI:** 10.1001/jamanetworkopen.2024.47617

**Published:** 2024-11-25

**Authors:** Derrick L. Tao, Mirella Nardo, Cheuk Hong Leung, Heather Y. Lin, Lei Kang, Hung Le, Ecaterina E. Dumbrava, David S. Hong

**Affiliations:** 1 University of Texas MD Anderson Cancer Center, Houston

## Abstract

This cohort study investigates the association of obesity with adoptive cell therapy outcomes in patients with solid tumors.

## Introduction

The obesity paradox describes the phenomenon in which obesity is associated with increased cancer risk but improved survival in patients treated with immune checkpoint inhibitors.^1,2^ Research suggests that biological changes due to adiposity, like alterations in tumor metabolism, immune environment, and angiogenesis, may explain this paradox.^3,4^ Meanwhile, adoptive cell therapy (ACT) is being developed for solid tumors despite challenges with heterogeneous tumor responses and toxic effects. The association of obesity with the efficacy and safety of ACT in patients with solid tumors is unclear. This retrospective multicohort analysis investigates this association in patients treated in various ACT clinical trials.

## Methods

This cohort study was approved by the institutional review board of MD Anderson Cancer Center and followed the STROBE reporting guideline for cohort studies. All participants provided informed consent. We included all patients with solid tumors treated on an ACT protocol in the Department of Investigational Cancer Therapeutics over the past 5 years. Body mass index (BMI; calculated as weight in kilograms divided by height in meters squared) categories were defined as underweight (<18.5), normal weight (18.5-24.9), overweight (25-29.9), and obesity (≥30). Fisher exact test compared categorical variables, with pairwise comparisons and Bonferroni correction as needed; *t* tests compared continuous variables. Overall and progression-free survival were estimated using the Kaplan-Meier method, with hazard ratios from Cox proportional hazard regression models. A 2-sided *P* value <.05 was considered significant. Analyses were performed using SAS version 9.4 (SAS Institute) and R version 4.3.1 (R Project for Statistical Computing) statistical software.

## Results

From August 2017 to August 2023, 95 consecutive patients with advanced cancer (median [range] age at treatment, 60.9 [20.5-84.0] years; 54 female [56.8%]; 2 Asian [2.1%], 4 Black [4.2%], 1 Hispanic [1.1%], and 84 White [88.4%]) received a cellular therapy product on protocol at MD Anderson Cancer Center and were included (Table). There were 39 patients (41.1%) with normal weight, 26 patients (27.4%) with overweight, 27 patients (28.4%) with obesity, and 3 patients (3.2%) with underweight. The most prevalent cancers were gastrointestinal cancer (21.1%), head and neck cancer (16.8%), gynecologic cancer (15.8%), sarcoma (11.6%), mesothelioma (10.2%), and breast cancer (8.4%).

**Table.** Characteristics of Patients at Baseline

Characteristic	Patients, No. (%) (N = 95)
Sex	
Female	54 (56.8)
Male	41 (43.2)
Race and ethnicity^a^	
Asian	2 (2.1)
Black	4 (4.2)
Hispanic or Latino	1 (1.1)
White	84 (88.4)
Other	4 (4.2)
ECOG performance status	
0	16 (16.8)
1	79 (83.2)
No. of metastasis sites	
0	15 (15.8)
1-2	50 (52.6)
≥3	30 (31.6)
No. of prior treatments	
0-1	15 (15.8)
≥2	80 (84.2)
Steroid history	
No	57 (60.0)
Yes	38 (40.0)
Cancer diagnosis	
Gastrointestinal	22 (23.2)
Head and neck	16 (18.8)
Gynecologic	15 (15.8)
Sarcoma	11 (11.6)
Mesothelioma	10 (10.5)
Breast	8 (8.4)
Product type	
TCR-T	63 (66.3)
TRuC T	11 (11.6)
NK	9 (9.5)
CAR-T	8 (8.4)
TAC	4 (4.2)

Abbreviations: CAR-T, chimeric antigen receptor T; ECOG, Eastern Cooperative Oncology Group; NK, natural killer; TAC, T-cell antigen coupler; TCR-T, T-cell receptor-engineered T cell; TRuC, T-cell receptor fusion construct.

^a^ Race and ethnicity were assessed by self-report then input into the electronic health record when patients were assessed as part of intake procedures. All race and ethnicity options were listed on the same form as seen here.

The median follow-up was 39.9 months, with a median progression-free and overall survival of 2.8 months (95% CI, 2.3-3.5 months) and 8.4 months (95% CI, 6.4-11.0 months), respectively. The risk of death among patients with obesity was significantly greater than among those with normal weight (hazard ratio, 0.56; 95% CI, 0.31-0.99; *P* = .045) (Figure). No weight difference was found between those who did and did not respond.

**Figure.**
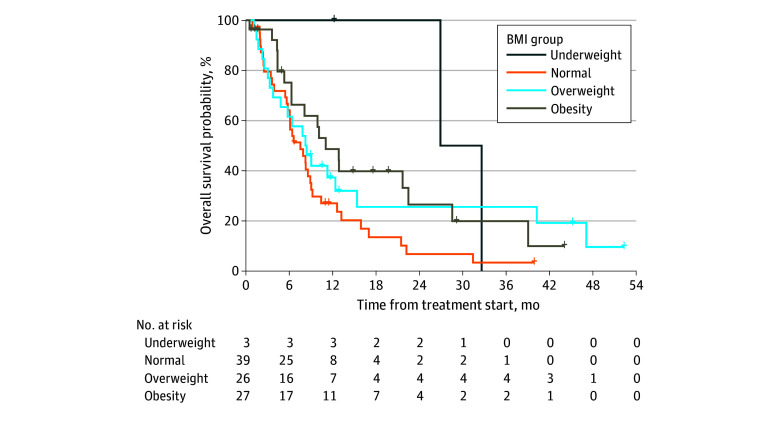
Kaplan-Meier Overall Survival Curves Overall survival is shown by body mass index (BMI; calculated as weight in kilograms divided by height in meters squared) group.

BMI was not associated with the development of cytokine release syndrome. However, patients with obesity had a lower rate of immune effector cell–associated neurotoxicity syndrome (ICANS) than those with normal weight (0% vs 23.1%; *P* = .049).

## Discussion

To the best of our knowledge, this cohort study is the first study evaluating the association of obesity with outcomes across multiple ACT products in a tumor-agnostic cohort. Studies of ACT in hematologic malignant cancers have not found consistent association between obesity and clinical outcomes.^5,6^ In our study, obesity was associated with improved survival and decreased ICANS rates after ACT. These results should be viewed as exploratory owing to study limitations, including heterogeneity and limited power for subgroup analysis. While BMI is a readily available measure, it does not fully capture obesity or other patient factors, such as visceral adiposity, muscle mass, or body composition. Further research is needed to understand how factors like genetics, socioeconomics, diet, exercise, and microbiota may contribute to outcomes among patients with obesity. To extend the benefits of novel immunotherapeutic strategies, such as ACT, to more patients with advanced solid tumors, it is crucial to understand factors associated with response and toxic effects. Immune-modifying factors, such as obesity, may be as significant as tumor-related factors in determining outcomes.

## Notes

Supplement.
Data Sharing Statement
