# Supplementary material for: Obesity and Outcomes in Adoptive Cellular Therapy in Solid Tumors
Source: JAMA Netw Open. 2024 Nov 25;7(11):e2447617. doi: 10.1001/jamanetworkopen.2024.47617 (PMC11589797; doi:10.1001/jamanetworkopen.2024.47617)
Supplement: Supplement. — Data Sharing Statement [file jamanetwopen-e2447617-s001.pdf]

Data Sharing Statement

Tao. Obesity and Outcomes in Adoptive Cellular Therapy in Solid Tumors. JAMA Netw Open. Published online November 25, 2024. doi:10.1001/jamanetworkopen.2024.47617

Data
Data available: No
